# Preoperative Optical Coherence Tomography Markers and Their Significance in the Treatment of Macular Holes Using the Inverted Internal Limiting Membrane Technique

**DOI:** 10.7759/cureus.79837

**Published:** 2025-02-28

**Authors:** Aleksandra Górska, Jan Osicki, Monika Bonczek, Joanna Rak, Sebastian Sirek, Dorota Pojda-Wilczek

**Affiliations:** 1 Department of Ophthalmology, Medical University of Silesia, Katowice, POL

**Keywords:** ftmh, macular hole, optical coherence tomography, ppv, predictors

## Abstract

Purpose

The purpose of this study was to evaluate the short-term outcomes of pars plana vitrectomy (PPV) using the inverted flap technique for full-thickness macular hole (FTMH). Specifically, the study aimed to identify significant preoperative optical coherence tomography (OCT) parameters that can serve as predictors for postoperative distance and near best-corrected visual acuity (DBCVA and NBCVA).

Methods

A prospective analysis was conducted on patients diagnosed with FTMH who underwent PPV from September 2022 to November 2024 using the inverted flap technique. OCT imaging was conducted preoperatively and one month postoperatively. Parameters analyzed included base diameter (BD), height of the macular hole (HT), right arm length (RAL) of macular hole (MH), left arm length (LAL) of MH, macular hole index (MHI), diameter hole index (DHI), tractional hole index (THI), hole form factor (HFF) and central retinal subfield (CRS). Statistical analysis was conducted to calculate significant predictors for DBCVA and NBCVA short-term postoperative results after PPV, with statistical significance set at p<0.05.

Results

A total of 46 patients, 35 (76.1%) women and 11 (23.9%) men, aged 67.6±5.7 were included in the study. Means and standard deviation for analyzed parameters were BD=937.5±355 𝜇m, HD=424.6±188 𝜇m, HT=413.6±56.5 𝜇m, RAL=359.6±125.5 𝜇m, LAL=367.5±214 𝜇m, MHI=0.477±0.12, DHI=0.46±0.15, THI=1.2±0.71, HFF=0.79±0.17 and CRS=297.4±21.8 𝜇m. Twenty-four patients presented with large FTMH (≥400 µm), and twenty-two with small FTMH (<400 µm). All patients achieved full closure of the FTMH. The most common comorbidity was hypertension, with 13 patients (28.3%). The only statistically significant predictor of DBCVA change after surgery was MHI (p=0.01).

Conclusions

PPV surgery using the inverted flap technique has very high efficiency. MHI can be used in the prediction of postoperative DBCVA. Further research is needed to assess the role of THI and HFF as other predictors.

## Introduction

A full-thickness macular hole (FTMH) is an anatomic defect in the retina that involves all layers, from the internal limiting membrane (ILM) to the photoreceptor layer [1]. Risk factors include high myopia, ocular trauma, macular telangiectasia type 2, wet age-related macular degeneration with intravitreal injections of anti-VEGF, and hypertension; however, it is usually idiopathic [2,3].

The incidence of macular holes (MH) in the general population ranges from 0.2% to 0.8% [4]. MH primarily affects people over 60 years old, and a high majority of them are women. In most cases, MH is unilateral; only 10-15% of patients have bilateral MH [3-5]. Symptoms such as decreased vision, metamorphopsia, and central dark spots can occur suddenly or gradually [4]. According to the International Vitreomacular Traction Study, MH is classified into three groups based on its shortest horizontal diameter on OCT: small (≤250 μm), medium (250-400 μm), and large (≥400 μm) [6].

The standard treatment approach for MH is pars plana vitrectomy (PPV), which is associated with a consistently high success rate exceeding 90%, as reported in numerous studies [3-6]. Despite the high effectiveness of anatomical closure in surgical treatment, full functional recovery is not always achieved [6]. For this reason, studies are being conducted to find factors that can predict the final outcomes of the procedure [1,5,6]. Previous researchers have confirmed the correlation between some preoperative OCT predictors and functional success [5,6].

## Materials and methods

Patients

A total of 46 patients, 35 women (76.1%) and 11 men (23.9%), with a mean age of 67.6±5.7 years, were included in the study. Inclusion criteria were as follows: a clinical diagnosis of FTMH confirmed via spectral-domain optical coherence tomography (SD-OCT), age above 18 years, and patient consent to adhere to the study protocol. Exclusion criteria included prior retinal surgery, other retinal disorders, significant media opacities impairing image quality, a history of uveitis or retinitis, ocular trauma, high myopia (≥-6.0 Dsph), hereditary or congenital retinal diseases, other ocular conditions affecting retinal function (e.g., intraocular tumors, post-intraocular foreign body conditions, or retinal vascular circulation disorders), anterior segment conditions preventing pupil dilation, retinal dystrophies or age-related macular degeneration, prior anti-VEGF therapy, connective tissue disorders, ongoing oncological or immunosuppressive treatment, prior hyperbaric therapy, and lack of patient consent.

Surgery and equipment

All patients underwent standard 23-gauge PPV performed by an experienced retinal surgeon. The surgery involved inducing posterior vitreous detachment, peeling the ILM with membrane blue, and gas tamponade using sulfur hexafluoride (SF6). Postoperative seven-day face-down positioning was recommended to promote MH closure. Optical coherence tomography (OCT) and OCT angiography (OCT-A) were conducted using the Optovue XR Avanti system (Visionix/Optovue Inc, North Lombard, USA). Scans were performed preoperatively and at one month postoperatively, covering a 6×6 mm area centered on the fovea.

Data

**Figure 1:**
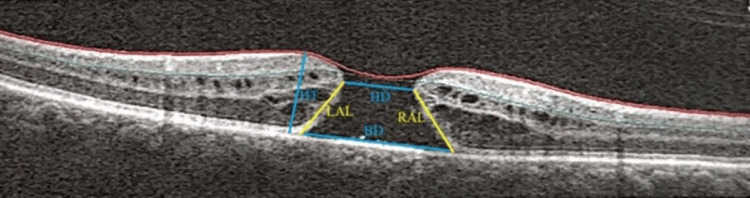
Measurements obtained from OCT scans: BD, HT, HD, LAL, and RAL BD: base diameter; HT: height of the macular hole; HD: narrowest distance in the macular hole; LAL: left arm length; RAL: right arm length

We conducted a single-center prospective cohort study at the Department of Ophthalmology, University Clinical Center, Medical University of Silesia in Katowice. We collected various data, including demographic and clinical information about patients' distance and near best-corrected visual acuity (DBCVA, NBCVA), measurements of MH from OCT and OCT-A scans, comorbidities, and the time from the onset of the disease to surgery. Distance visual acuity was measured using Snellen charts, and near visual acuity was assessed using Jaeger charts. Measurements from OCT scans included base diameter (BD), the height of the macular hole (HT), the narrowest distance in the macular hole (HD), left arm length (LAL) of MH, and right arm length (RAL) of MH. Measurements are shown in Figure 1. Based on the collected data, we calculated relevant predictive factors for BCVA improvement, as known from published literature, and changes in both distance and near visual acuity [4,5,6]. Calculated predictors included macular hole index (MHI)=HT/BD, diameter hole index (DHI)=HD/BD, tractional hole index (THI)=HT/HD, and hole form factor (HFF)=(LAL + RAL)/BD. Registered comorbidities included diabetes, coronary heart disease (CHD), and hypertension.

Through a systematic evaluation of these parameters, the research aimed to provide detailed insights into the microvascular changes in the retina that occur following vitrectomy for FTMH.

Statistical analysis

Continuous variables with a normal distribution were expressed as mean ± standard deviation (SD), while those not following a normal distribution were reported as median (ME) with interquartile range (IQR). Categorical variables were summarized as frequencies and percentages. The Shapiro-Wilk test was used to assess the normality of the data distribution. Statistical analyses were performed using appropriate methods based on the distribution type. Group differences based on sex, affected eye, presence of comorbidities, and continuous variables were analyzed using the Student’s t-test or Mann-Whitney U test, as appropriate. Correlations between continuous variables were evaluated using Pearson’s correlation coefficient or Spearman’s rank-order coefficient. A p-value <0.05 was considered statistically significant for all analyses. Data analysis was performed using licensed statistical software (Statistica 14: TIBCO Software Inc. Palo Alto, US).

Ethics

The study was conducted in accordance with the principles outlined in the Declaration of Helsinki and received approval from the Bioethics Committee of the Medical University of Silesia (PCN/CBN/0052/KB1/122/22). Written informed consent was obtained from all participants prior to enrollment.

## Results

A total of 46 patients were included in the study. Twenty-four patients presented with large FTMH (≥400 μm), and 22 with small FTMH (<400 μm). All patients achieved full closure of the FTMH. The most common comorbidity was hypertension, with 13 patients (28.3%). There were no differences between the groups in terms of age, sex, eye, or comorbidities, including CHD, diabetes, and hypertension. Patients' distance and near visual acuity were measured before surgery and one month after surgery on the decimal scale (Table 1).

**Table 1:** Values of visual acuity (decimal scale) ME: Median; DBCVA: distance best-corrected visual acuity; NBCVA: near best-corrected visual acuity; FTMH: full-thickness macular hole: PPV: pars plana vitrectomy

	ME	Min.	Max.	Q1 (25^th^ percentile)	Q3 (75^th^ percentile)
DBCVA FTMH eye before PPV	0.08	0.02	0.4	0.04	0.5
DBCVA FTMH eye one month after PPV	0.3	0.02	0.9	0.1	0.4
DBCVA fellow eye before PPV	1.0	0.7	1.0	0.9	1.0
NBCVA FTMH eye before PPV	2.25	0.5	3.0	1.5	3.0
NBCVA FTMH eye one month after PPV	0.75	0.5	3.0	0.5	1.25
NBCVA fellow eye before PPV	0.5	0.5	0.75	0.5	0.5

The BD exhibited a ME of 944.5 μm. The central retinal subfield (CRS) showed a ME thickness of 293 μm. The HD had a ME of 412.5 μm. The HT showed a mean of 413.65 μm. Indices such as the DHI, MHI, and HFF were characterized by ME values of 0.46, 0.46, and 0.77, respectively, with varying ranges and standard deviations provided. The THI presented a ME of 1.04, ranging from 0.36 to 4.6 (Table 2).

**Table 2:** Measured morphological parameters and their corresponding calculated predictors BD: base diameter; CRS: central retinal subfield; HD: narrowest distance in macular hole; HT: height of macular hole; DHI: diameter hole index; MHI: macular hole index; THI: tractional hole index; HFF: hole form factor; ME: median

	Mean	ME	Min.	Max.	Lower quartile	Upper quartile	Standard deviation
BD (μm)	-	944.5	442	2560	706	1070	-
CRS (μm)	-	293	261	376	286	307	-
HD (μm)	-	412.5	129	1040	271	542	-
HT (μm)	413.65	407.5	304	593	-	-	56.52
DHI	0.46	0.46	0.1	0.84	-	-	0.15
MHI	0.48	0.46	0.17	0.74	-	-	0.13
THI	-	1.04	0.36	4.6	0.81	1.39	-
HFF	0.79	0.77	0.49	1.17	-	-	0.17

The only statistically significant predictor of distance visual acuity change after surgery was MHI (p=0.01). There were no statistically significant correlations between any other morphological factors, the calculated predictors considered, and changes in distance and near visual acuity. There were no statistically significant differences between groups based on age, sex, eye, diabetes, CHD, or hypertension.

## Discussion

The development of surgical techniques has enabled MH surgery using the inverted flap technique to achieve an effectiveness rate of 98% [7]. After the FTMH is closed, the outer retinal layers (EML, ONL, EZ) regenerate, which is accompanied by a gradual improvement in visual acuity [5-11]. These studies consistently report that visual acuity improves from a baseline of approximately 20/200 to a postoperative range of 20/50 to 20/30, depending on the study and patient population. This process is long-term; according to the available literature, it lasts at least three years [8]. Therefore, numerous studies are being conducted to evaluate the role of preoperative OCT parameters in predicting both short-term and long-term best-corrected visual acuity (BCVA) [6,9,10,11].

BD is defined as the linear dimension of the MH at the level of the retinal pigment epithelium layer [6]. Its importance in predicting the outcome of surgery is controversial. S. Ullrich et al. showed that BD, in particular, seems to be a valuable predictor of MH surgery [12]. A retrospective analysis by Laura Wakely et al. demonstrated that as the BD of an MH increases, the probability of achieving both anatomical and visual success diminishes [10]. However, Shilpi H. Narnaware et al. did not show any correlation between BD and postoperative visual acuity [6]. The results of our study did not reveal significant differences between BD and the change in distance visual acuity.

The minimum linear diameter is the narrowest diameter of the hole in the approximate central part of the retina [13]. In a prospective cohort study conducted in the UK between 2015 and 2018 by DH Steel et al., the procedure success rate was 97%-98% for minimum linear diameter values <500 μm, 90% for holes of 500-599 μm, and 87% for holes of >600 μm [5].

The HT is defined as the maximum distance between the vitreoretinal junction of the hole and the retinal pigment epithelium. The study by Christos Haritoglou et al. showed a negative correlation between HT and final visual acuity, but other analyses conducted in the past did not show such an association [6,14]. Our study also did not confirm the existence of such a relationship.

The HFF is the ratio of the sum of the lengths of the left and right arms of the MH and the BD. In the past, it was considered one of the OCT predictors of the effectiveness of MH closure, but with the introduction of the inverted flap technique as a routine surgical technique, it lost its significance, which was also confirmed by our study [6].

The MHI is a ratio of the hole height to the BD of the MH [15]. According to Kusuhara et al., there is a significant correlation between MHI and postoperative BCVA [15]. Retrospective analysis by Bajdik et al. suggests that MHI can also be a useful predictor of short-term BCVA [16]. However, a study conducted by Narnaware et al. did not reveal any statistically significant correlation between MHI and visual acuity gain [6]. In our study, MHI was the only statistically significant predictor in prognosing distance visual acuity change after surgery.

The DHI was defined by JM Ruiz-Moreno et al. as the ratio of the minimum diameter of the MH to the BA [9]. However, the analysis did not reveal any significant correlation between DHI and postoperative BCVA. Research conducted by Bajdik et al. also did not show any significant correlation between DHI and postoperative visual acuity [16].

The THI is the ratio of the maximal height to the minimum diameter of the MH. The retrospective case series study by JM Ruiz-Moreno et al. did not reveal any significant correlation between THI and postoperative best spectacle-corrected visual acuity at three months [9]. The results of our research also did not show any significant correlation between THI and postoperative visual acuity.

In a study comparable to ours, a longer follow-up period of at least six to 12 months would be necessary to gain a more comprehensive understanding of the healing process and potential long-term outcomes [17-19]. Studies such as those by Purtskhvanidze et al. and Leonard et al. have demonstrated that visual acuity and anatomical recovery stabilize within six to 12 months post-surgery, with further improvements observed up to 24 months in some cases [17,19]. Extending the follow-up period would allow for a more detailed assessment of functional and anatomical outcomes, particularly in relation to outer retinal layer regeneration and long-term visual stability. Previous research has shown that improvements in visual acuity can persist for up to two years and tend to stabilize after a longer period [17-19]. However, some studies suggest that healing times may vary depending on the size of the FTMH, with visual improvement potentially occurring later than the one-month follow-up period that we examined. Additionally, MH chronicity (time-lapse from FTMH onset to surgery) may play a significant role in the rate of BCVA recovery. For instance, Bleidißel et al. reported that longer-standing MHs (>6 months) showed delayed visual improvement and slower regeneration of the outer retinal layers compared to acute cases (<3 months). This variability in recovery rates based on chronicity could pose a limitation to our study outcomes, as our one-month follow-up period may not have fully captured the extent of visual improvement in chronic cases. Future studies should consider stratifying patients based on chronicity to provide a more comprehensive understanding of recovery patterns [20]. While our study focused on data collected preoperatively and one month after surgery, we plan to conduct further follow-up assessments to capture long-term recovery trends. Additional data over extended periods would help us better understand the healing trajectory of different types and sizes of MH, as well as identify other potential predictors that may influence visual recovery over time.

## Conclusions

PPV surgery using the inverted flap technique is characterized by very high efficiency in closing MHs and is associated with significant improvement in visual acuity.

MHI is the only useful factor in predicting short-term postoperative BCVA. There is no significant correlation between other parameters measured in OCT and postoperative BCVA; however, further research is needed to assess the role of THI and HFF as additional predictors.
